# Total Polyphenol Content and Other Antioxidant Capacity Indicators in Selected Edible Plants and Herbs

**DOI:** 10.3390/molecules31142523

**Published:** 2026-07-20

**Authors:** Katarzyna Aleksandra Kaczmarek-Kryszak, Patryk Mizera, Grzegorz Kosewski, Lidia Szwajkowska-Michałek, Katarzyna Rzyska-Szczupak, Anna Szczepańska-Álvarez, Kinga Stuper-Szablewska, Krzysztof Dziedzic, Sławomira Drzymała-Czyż

**Affiliations:** 1 Department of Bromatology, Poznan University of Medical Science, Rokietnicka 3 Street, 60-806 Poznan, Poland; grzegorzkosewski@ump.edu.pl (G.K.);; 2 Doctoral School, Poznan University of Medical Sciences, Bukowska 70 Street, 60-812 Poznan, Poland; 3 Department of Food Technology of Plant Origin, Faculty of Food Science and Nutrition, Poznan University of Life Sciences, Wojska Polskiego 31, 60-624 Poznan, Polandkrzysztof.dziedzic@up.poznan.pl (K.D.); 4 Department of Chemistry, Faculty of Forestry and Wood Technology, Poznan University of Life Sciences, Wojska Polskiego 75, 60-625 Poznan, Polandkatarzyna.rzyska@up.poznan.pl (K.R.-S.);; 5 Department of Mathematical and Statistical Methods, Poznan University of Life Sciences, Wojska Polskiego 28, 60-637 Poznan, Poland; anna.szczepanska-alvarez@up.poznan.pl

**Keywords:** polyphenols, flavonoids, anthocyanins, oxidative stress, antioxidants

## Abstract

Oxidative stress and antioxidant enzyme activity play a major role in the development of numerous chronic diseases. Edible plants and herbs are rich in polyphenols and other bioactive compounds that may protect cells by neutralizing free radicals and supporting endogenous defense systems. This study compared the antioxidant potential of 10 plant extracts using seven analytical methods. The total polyphenol, flavonoid, and anthocyanin contents were measured, while the antioxidant activity was assessed with DPPH, ABTS, FRAP, and CUPRAC assays. The statistical analyses included an ANOVA and a principal component analysis (PCA). Significant differences were observed among the extracts (*p* < 0.001). *P. rotundifolia* showed the highest polyphenol content and the strongest FRAP and ABTS activity. The greatest flavonoid levels were found in *C. sativus* and *A. crassna*, whereas *E. cardamomum* contained the highest anthocyanin levels. A strong correlation was observed between the total polyphenol content and the FRAP results, while the anthocyanin content showed an inverse relationship with the DPPH activity. The findings confirm the high antioxidant potential of the studied plants and suggest their promising application in the development of alternative and complementary therapies in the course of chronic diseases.

## 1. Introduction

### 1.1. Antioxidative Properties of Plant-Derived Compounds

Polyphenols are the widest group of plant-derived organic compounds. They possess a very high antioxidative potential, which works both directly and indirectly. The direct mechanism of action is based on the neutralization of reactive oxygen species. Antioxidant activity is closely linked to the structure of the molecule—the presence of hydroxyl groups on aromatic rings is crucial for the ability to donate electrons, which can balance free radicals and limit their reactivity. This ultimately reduces oxidative stress and prevents the destruction of DNA, proteins, and lipids [1,2]. The indirect mechanism of action is determined by the stimulation of antioxidative enzymes through the NRF2/ARE pathway. It eventually increases the levels of glutathione peroxidase, glutamate-cysteine ligase, glutathione S-transferases, NAD(P)H dehydrogenase, and heme oxygenase 1 [3].

Flavonoids are a subgroup of polyphenols, specifically aromatic amino acids. The mechanism of flavonoid antioxidant activity is complex and involves both the direct neutralization of free radicals and interactions with various enzymatic systems in the body. As exogenous antioxidants, flavonoids are directly oxidized by free radicals, thereby forming less reactive, more stable chemical forms [4]. Flavonoids reduce oxidative stress by inhibiting nitric oxide synthase (NOS) and xanthine oxidase activity, which limits the formation of new free radicals. They also modulate cellular signaling pathways and interact with other enzymatic systems. Flavonoids possess the ability to chelate metal ions, which act as oxidation catalysts [5]. All of the above result in a strong antioxidative potential.

**Figure 1:**
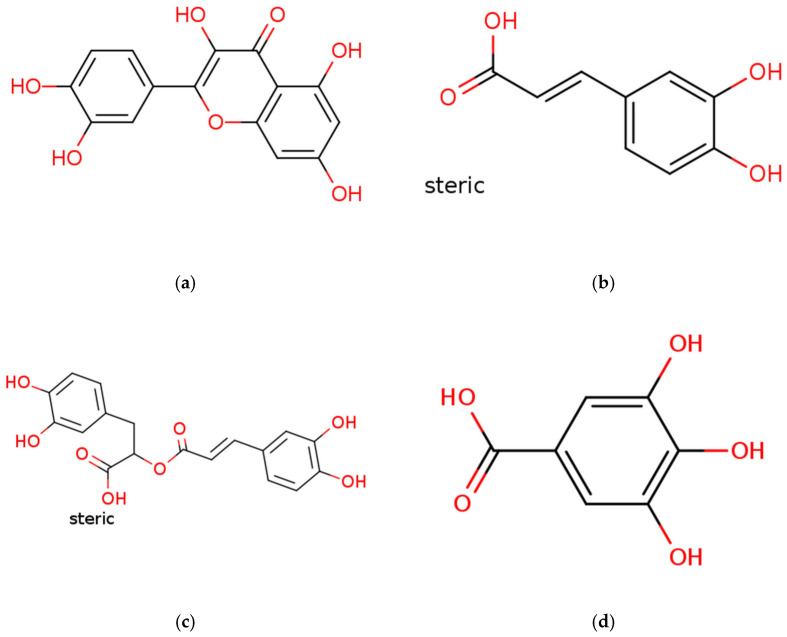
Chemical structure of quercetin (**a**), caffeic acid (**b**), rosmarinic acid (**c**), and gallic acid (**d**).

In the example of four widely present plant-derived phenolic compounds—quercetin, caffeic acid, rosmarinic acid, and gallic acid—the strong antioxidant properties can be chemically explained (Figure 1). Quercetin possesses two benzene rings (external) and one pyron ring (middle). The two hydroxyl groups (-OH) attached to the benzene ring create a catechol group, which gives the molecule the ability to easily donate electrons, neutralizing free radicals. Additionally, the hydroxyl (-OH) and ketone (=O) groups in the middle ring confer a strong ability to chelate metal ions [6].

The caffeic acid molecule contains a catechol group as well, which provides its antioxidant potential. The additional acrylic acid side chain (-CH=CH-COOH) makes the molecule more stable after electron donation [7].

Rosmarinic acid contains two full catechol groups and an additional carboxyl group, which more than doubles its electron-donating capacity. The presence of a double bond (C=C) conjugated with the aromatic ring stabilizes the molecule after electron donation, thus making the resulting phenoxyl radical less reactive [8].

Gallic acid possesses three hydroxyl groups (-OH) that are densely arranged. Additionally, the molecule contains a carboxyl group, providing another -OH to increase the reductive potential [9].

Anthocyanins are a class of flavonoids that are responsible for reds and blues in plants. The antioxidant mechanism of action of anthocyanins is based on two main pillars: the direct neutralization of free radicals and the indirect influence on cellular defense systems. Free radical scavenging can be achieved in two ways: hydrogen atom transfer (HAT)—a free radical removes a hydrogen atom from the anthocyanin molecule, transforming it into a more stable product—and single electron transfer (SET)—an anthocyanin donates an electron to the free radical. As a result, aroxyl radicals are formed, which, due to the resonance phenomenon, are much more stable than the radicals they reduced, which allows for the effective interruption of oxidative chain reactions. Due to the low bioavailability of anthocyanins, their direct effects in the body are limited. Their indirect effects play a key role, stimulating the cell’s own defense mechanisms. Anthocyanins stimulate the biosynthesis of antioxidant proteins by activating the Nrf2/ARE signaling pathway. Their presence also contributes to the increased level or protection of key enzymes, such as superoxide dismutase, catalase, and glutathione peroxidase. Anthocyanins can inhibit proinflammatory factors such as NF-κB, indirectly reducing the oxidative stress associated with inflammation.

### 1.2. The Role of Oxidative Stress in the Pathogenesis of Health Disorders

In a state of homeostasis, the generation of reactive oxygen species (ROS) is balanced with the activity of antioxidative enzymes and proteins, such as superoxide dismutase, catalase, or glutathione. A disproportion in these two matters creates a state of oxidative stress, which can negatively influence the cell by organelle damage, mitochondrial damage, and DNA disruption [10,11]. Surpassing the oxidative threshold can lead to the production of proapoptotic proteins, and later, induce cell apoptosis [12].

In the case of the insufficient presence of antioxidative agents, they can be provided using a diet. Edible plants (fruits, vegetables, herbs) are known to contain high levels of antioxidant compounds, which can act as preventative measures from numerous diseases. An anti-inflammatory diet has been described as efficient in multiple disorders, for example, depression, neurodegenerative disorders, and other chronic diseases. Since 2015, there has been an officially developed dietary intervention called MIND (Mediterranean–DASH Intervention for Neurodegenerative Delay) [13]. It aims to minimize the risk of developing neurodegenerative disorders, mainly by increasing the intake of leafy green vegetables and berries, which are abundant in antioxidative polyphenols. The satisfactory results of the MIND diet advocate for a great potential of dietary polyphenols and antioxidants in human health, especially the central nervous system.

### 1.3. Aims and Hypothesis

We hypothesize that the 10 species of edible plants and herbs studied demonstrate statistically and functionally significant differences in antioxidant potential, resulting from their unique phytochemical profiles. It is assumed that differences in the content of polyphenols, flavonoids, and anthocyanins directly determine the hierarchy of the effectiveness of these plants in different measurement systems, with the highest density of hydroxyl groups predisposing selected extracts to dominate metal ion reduction (SET) mechanisms, while specific structural features (e.g., the presence of glycosides) differentiate their strength in scavenging neutral and cationic radicals.

The aim of this study was to multidimensionally evaluate 10 plant extracts (*Pyrola rotundifolia*, *Zataria multiflora*, *Melissa officinalis*, *Elettaria cardamomum*, *Salvia officinalis*, *Aquilaria crassna*, *Ginkgo biloba*, *Crocus sativus*, *Paeonia radix*, and *Fragaria x ananasa*) using seven independent analytical methods. The ten plant species were selected based on criteria established in our prior systematic reviews [14,15], which identified them as priority candidates for comprehensive antioxidant profiling. Specifically, these species met the following selection criteria: (i) in vitro data supporting a neuroprotective potential through an enhanced neuronal cell viability in models of induced cytotoxicity, and (ii) human clinical data demonstrating improved cognitive function following the administration of plant extracts or isolated bioactive compounds. This study allows for an objective comparative assessment of the antioxidant capacity of the selected plants.

## 2. Results

**Figure 2:**
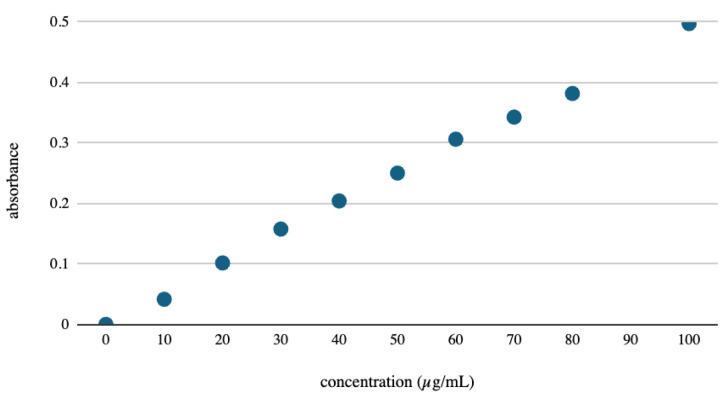
Calibration curve for TPC assessment.

**Figure 3:**
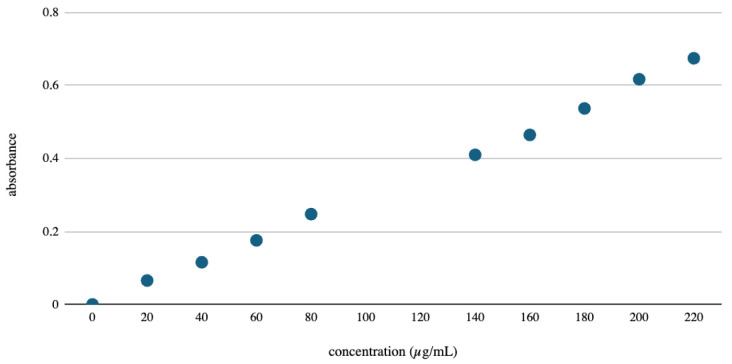
Calibration curve for TFC assessment.

**Figure 4:**
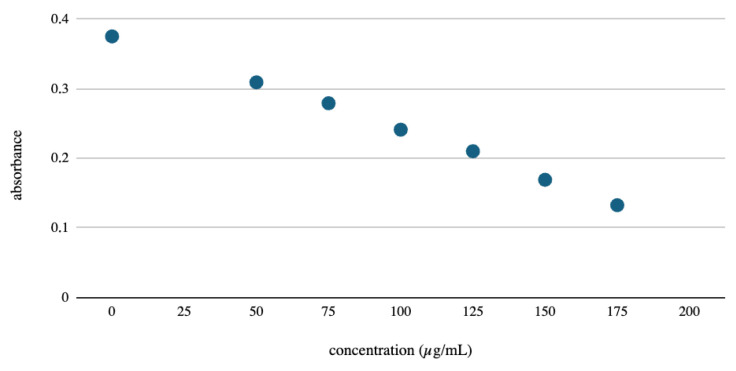
Calibration curve for DPPH assessment.

**Figure 5:**
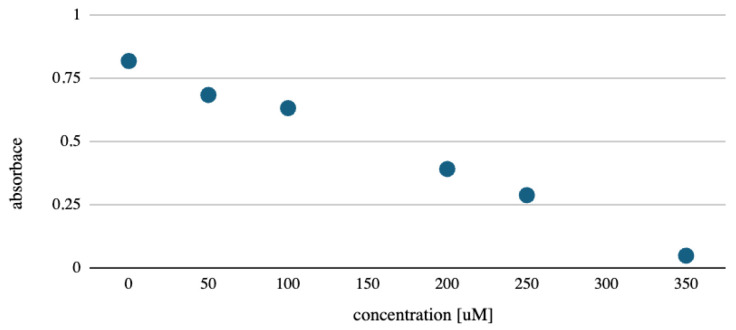
Calibration curve for ABTS assessment.

**Figure 6:**
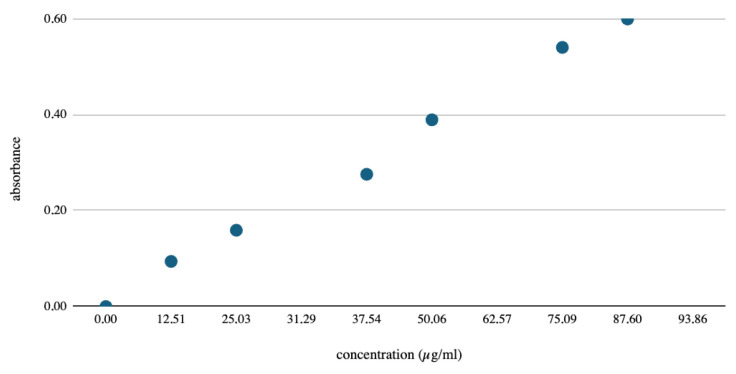
Calibration curve for FRAP assessment.

**Figure 7:**
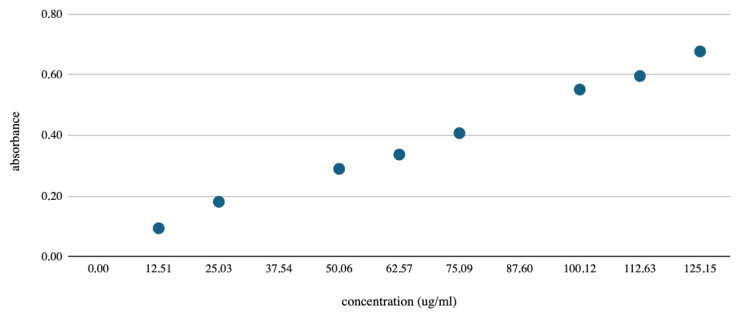
Calibration curve for CUPRAC assessment.

The results of all the conducted tests are shown in Table 1. In the case of total polyphenol content (TPC), the highest score was noted for *P. rotundifolia*. The highest total flavonoid content (TFC) was found for *C. sativus*, and the highest total anthocyanin content (TAC) for *E. cardamomum*. *P. rotundifolia* also achieved the highest scores in the FRAP and ABTS. In DPPH radical reduction, the best-scoring plant was found to be *S. officinalis*, and in the CUPRAC, *M. officinalis*. Calibration curves for each assessment are presented in Figure 2, Figure 3, Figure 4, Figure 5, Figure 6 and Figure 7.

There were highly significant differences between the TPC scores (F = 665, *p* < 0.001). *P. rotundifolia* showed the highest values, and *Z. multiflora* and *A. crassna* were also statistically significant leaders (*p* < 0.05).

The TAC was the only parameter where *P. rotundifolia* did not dominate. *E. cardamomum* exhibited a superior performance; it had significantly more anthocyanins than most samples, including saffron (*C. sativus*), ginkgo (*G. biloba*), and strawberry (*F. x ananasa*) (*p* < 0.05).

The global comparison of the results showed significant differences between the plants’ TFC scores (*p* < 0.005). However, pairwise comparisons did not show specific pairs with statistically significant differences. The most probable reason is the high internal variability and small sample size. Additionally, the applied test (Dwass–Steel–Critchlow–Fligner pairwise comparison) assigns statistical significance very carefully to avoid type I errors.

We found very strong differences (F = 258, *p* < 0.001) between the plants’ FRAP scores. *P. rotundifolia* had a significantly higher reduction potential than all other plants (mean differences ranging from 17.6 to 41.17 mg TE/g DPM, always *p* < 0.001).

Comparison of the ABTS results indicate significant differences between the groups. *P. rotundifolia* again outperformed the rest of the group, showing the greatest difference from cardamom (102.6 mg TE/g DPM, *p* < 0.001).

The global comparison models showed significant differences between samples in both DPPH (*p* < 0.005) and the CUPRAC (*p* < 0.002). Yet, pairwise comparisons did not reveal specific pairs with a strong statistical significance of difference (*p*-values typically > 0.5), suggesting that the differences between specific extracts are more difficult to resolve.

There was a significant correlation between the FRAP scores and the TPC and TFC. We found no significant correlations between other antioxidant capacity tests and the TPC, TFC, or TAC (Table 2).

Table 3 shows how individual variables influence the three main components (PCA):

Component 1: This axis is most strongly defined by the TPC (0.947) and FRAP (0.936), which means that this axis primarily represents the total polyphenol content and iron-reducing capacity. Based on this, the *P. rotundifolia* sample, which had the highest scores in these categories, was at the extreme positive end of the C1 axis.

Component 2: This axis has a very strong negative loading for anthocyanins (−0.950) and a strong positive loading for DPPH (0.917). This is a key finding, as it shows that, in our study, a high anthocyanin content does not necessarily correlate with a high DPPH radical-scavenging capacity. This is perfectly illustrated by *E. cardamomum*, which had the highest anthocyanins (0.4639 mg/g DPM), but almost zero DPPH (0.24 mg TE/g DPM).

Component 3: This component is dominated by the TFC (0.906), i.e., flavonoids, and it separates flavonoid-rich samples, such as *C. sativus* and *A. crassna*, from the other samples.

Of note is the CUPRAC parameter, which has a very high uniqueness value (0.7488). This indicates that the variability in the CUPRAC test results is largely independent of the other tests and is not fully explained by the three principal components. This may be due to the fact that *M. officinalis* achieved an extremely high, distinctive result in this test (136.81 mg TE/g DPM).

**Figure 8:**
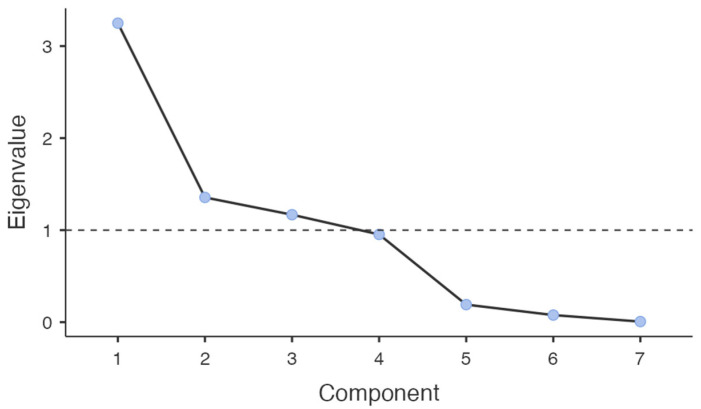
Scree plot of PCA.

Figure 8 and Table 4 justify the choice of the number of components for the PCA. The first three components have an eigenvalue above 1, which means that these three components are sufficient to explain the vast majority of the information contained in all seven tests. The subsequent components (4–7) contribute very little new knowledge to the model.

**Figure 9:**
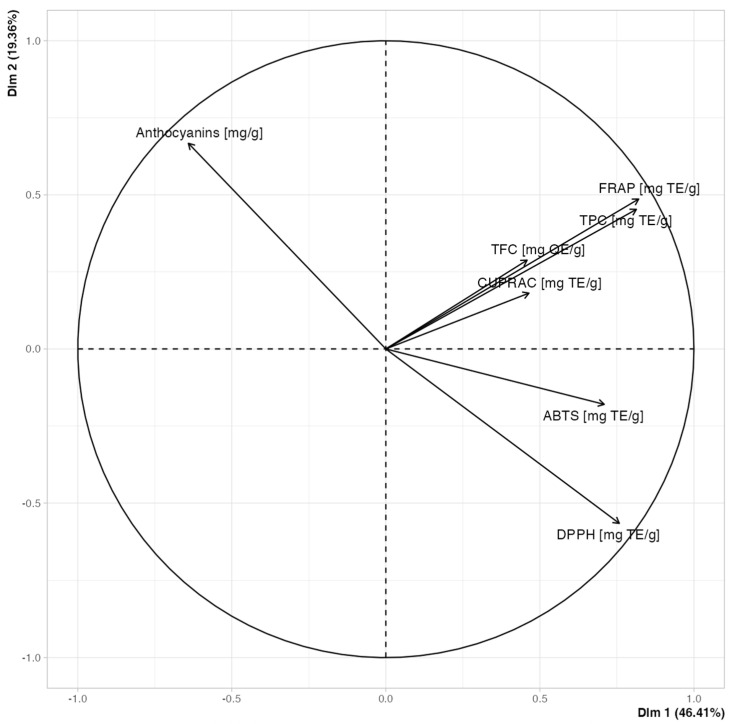
Correlation circle.

Figure 9 is a correlation circle, which translates the numbers from the scree plot into relationships. The first dimension (Dim 1), which has the highest value on the scree plot, explains 46.41% of the variability and is defined by the strongly correlated TPC and FRAP. The second dimension (Dim 2), corresponding to the next point on the scree plot, explains 19.36% of the variability. The correlation wheel shows that this axis primarily represents the opposition between anthocyanins and the DPPH activity. Because the first two dimensions (illustrated on the wheel) together explain nearly 66% of the total variance, we can confidently infer the antioxidant profile of plants based on their position relative to the vectors on the graph.

To analyze the extracts more thoroughly, we conducted an HPLC analysis of their phenolic compounds (Table 5 and Table S1). The most common compound groups were quercetin derivatives and caffeic acid derivatives. *Melissa officinalis* was distinguished by a very high content of rosmarinic acid derivatives (327.5 mg/g DE). *Aquilaria crassna* exhibited a uniquely high concentration of mangiferin derivatives (376.2 mg/g DE). *Paeonia radix* was rich in iridoids (157.6 mg/g DE), while *Fragaria x ananasa* contained significant amounts of ellagic acid derivatives (145.7 mg/g DE) and anthocyanins (80.5 mg/g DE). *Pyrola rotundifolia*, which dominated the antioxidant potential tests, contained mainly quercetin and gallic acid derivatives.

**Figure 10:**
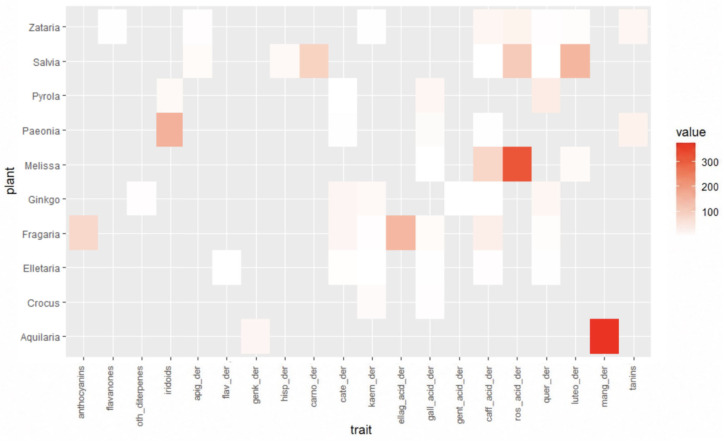
Heat map of the relationship between the analyzed plants and the derivatives of compounds determined in them.

Figure 10 provides a graphical visualization of the data in Table 5 and Table S1, showing the relationships between specific plant types and groups of derived chemical compounds.

**Figure 11:**
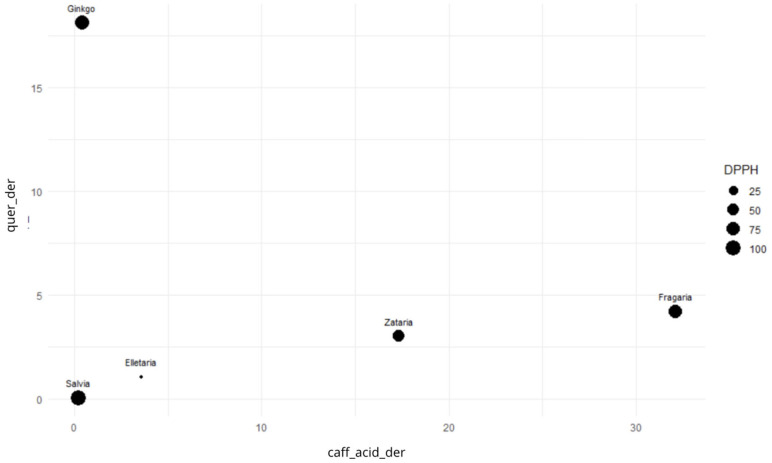
DPPH values depending on quercetin derivatives and caffeic acid derivatives.

**Figure 12:**
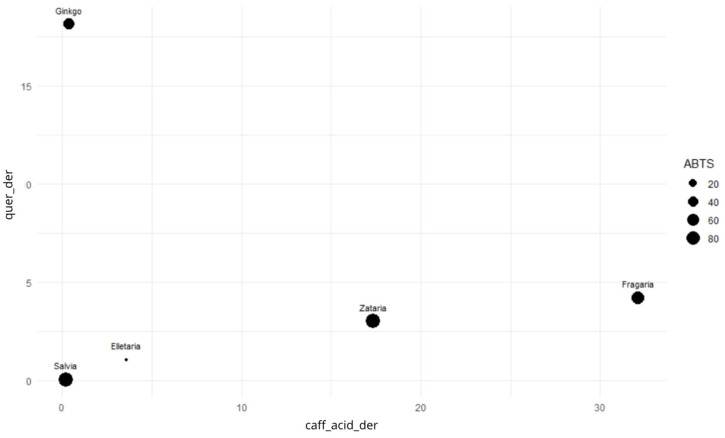
ABTS values depending on quercetin derivatives and caffeic acid derivatives.

**Figure 13:**
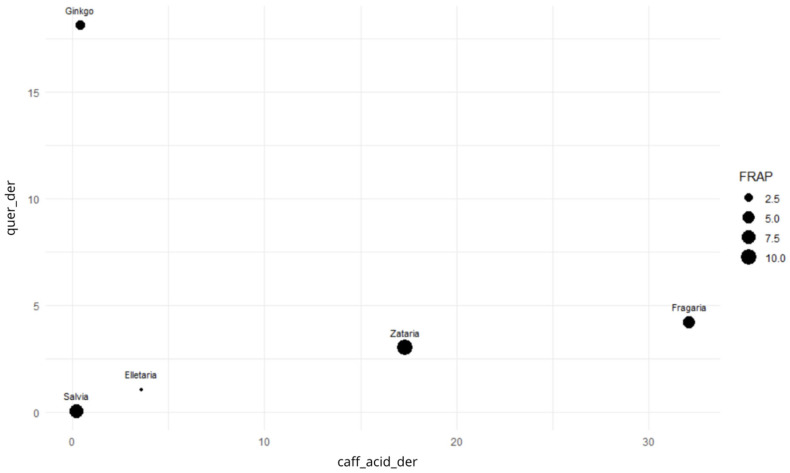
FRAP values depending on quercetin derivatives and caffeic acid derivatives.

**Figure 14:**
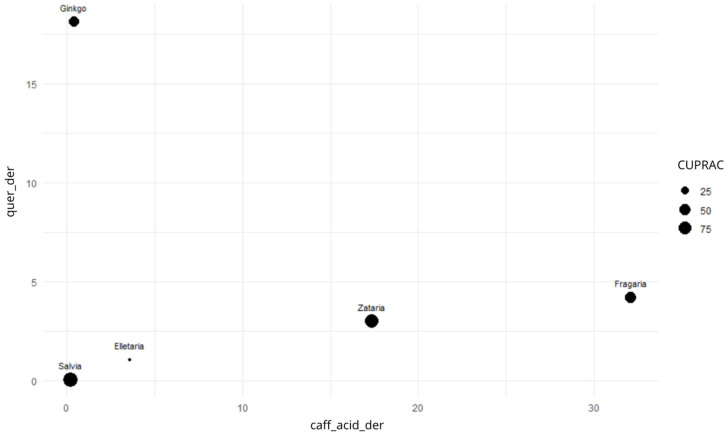
CUPRAC values depending on quercetin derivatives and caffeic acid derivatives.

Due to the fact that the most common groups of compounds determined in the extracts are quercetin derivatives (six out of 10 plant species) and caffeic acid derivatives (seven out of 10 plant species), we determined the values of the FRAP, ABTS, DPPH, and CUPRAC depending on their concentrations (Figure 11, Figure 12, Figure 13 and Figure 14). These graphs allow for an assessment of the extent to which these specific polyphenol groups are responsible for the measured antioxidant potential in different chemical systems.

## 3. Discussion

The results of this systematic review shed new light on the importance of incorporating a variety of edible plants and herbs into the diet, which are valued in medicine primarily for their high antioxidant potential. Plant-derived substances found in common foods such as strawberry fruit (*Fragaria* sp.) or cardamom seeds (*Elettaria cardamomum*) demonstrate the ability to effectively mitigate oxidative stress. The potential of these phytochemicals to reduce reactive oxygen species and stimulate endogenous defense mechanisms, such as increased glutathione levels, provides a crucial foundation for their pro-regenerative and neuroprotective effects. In the context of discussions on new therapeutic methods, it is these multivalent properties of edible plants, which go beyond simple antioxidant activity, that open up promising prospects for the use of phytotherapy and nutrition to support nervous system regeneration.

We found a strong correlation between the TFC, TPC, and FRAP scores. This relationship stems directly from the chemical mechanisms involved in these analyses and the molecular structure of the compounds tested. The FRAP test measures the extract’s ability to donate electrons, which allows for the reduction of iron ions (Fe^3+^) to (Fe^2+^) [16]. Both polyphenols and flavonoids act as strong reducing agents through the single electron transfer (SET) mechanism, and a larger number of hydroxyl groups (-OH) in their aromatic rings directly increases their electron-donating capacity, which translates into a higher FRAP test result [17]. The same hydroxyl density principle is the key predictor of the ABTS results.

This chemical profile best explains the dominance of *Pyrola rotundifolia,* which contains primarily quercetin derivatives and gallic acid derivatives. Quercetin has a catechol structure, and gallic acid has a pyrogallol structure; both structures are characterized by a high electron-donating potential, directly explaining *P. rotundifolia*’s highest scores in the FRAP and ABTS assays (confirmed by the strong positive correlation between the TPC and FRAP). The presence of an ortho-dihydroxyl (catechol) structure in the B ring ensures the high stability of the resulting radical, and three hydroxyl groups further enhance the scavenging capacity compared to catechol alone. *Aquilaria crassna*, with its uniquely high content of mangiferin derivatives, similarly shows a high ranking in the overall antioxidant activity ranking, supporting the same structure–activity relationship.

The higher FRAP values observed in extracts richer in quercetin can be explained by the chemical structure of this compound. The FRAP assay measures the ability of antioxidants to reduce Fe^3+^ to Fe^2+^ under acidic conditions, and quercetin possesses several structural features associated with a strong reducing capacity; multiple hydroxyl groups, a C2=C3 double bond conjugated with a 4-oxo group, and an extended system of electron delocalization. These features facilitate electron donation and stabilization of the oxidized radical form. However, quercetin should not be considered the only factor responsible for the FRAP performance in complex extracts—the assay results depend on the overall composition of the extract, the concentration of individual compounds, structural features, interactions between compounds, and assay-specific reaction conditions [18].

Our dose-dependance analysis of the FRAP, ABTS, DPPH, and CUPRAC against the quercetin and caffeic acid derivative content reinforces the picture. The FRAP shows the strongest positive relationship with these compounds: both caffeic acid derivatives (polyphenols) and quercetin (flavonoids) act as strong reducing agents via SET, and the higher their concentration—particularly evident in *P. rotundifolia*—the greater the ability to reduce Fe^3+^ to Fe^2+^. ABTS shows a similarly strong relationship for a related, but distinct, reason: as a cation radical (ABTS∙+), it reacts more readily than DPPH with large glycosidic molecules, such as quercetin derivatives.

One of the most interesting findings of this study is the strong negative correlation between the anthocyanin content and the DPPH activity—the clearest opposition captured in PCA Component 2. This is best illustrated by *Elettaria cardamomum*, which has the highest anthocyanin content in the dataset, but an almost zero DPPH radical-scavenging capacity, despite also containing caffeic acid derivatives and catechins.

Anthocyanins occur primarily as glycosides. We propose that these complex structures create a physical barrier (steric hindrance) that impedes contact between the antioxidant and the DPPH radical. The DPPH radical is neutral and particularly sensitive to this phenomenon, whereas the cationic ABTS radical, with different reaction kinetics, reacts far much more readily with large glycoside molecules [19]. This asymmetry explains why the anthocyanin content predicts low DPPH activity without predicting low ABTS activity [20]. Vectors pointing in opposite directions in the PCA suggest that, as the anthocyanin content increases in a sample set, the DPPH activity typically decreases, and vice versa. Based on this separation, we can hypothesize that the compounds responsible for the high anthocyanin content in these samples are not the primary DPPH-scavenging agents.

This finding should, however, be interpreted with caution and should not be read as evidence that anthocyanins reduce the antioxidant activity. The total anthocyanin content assay provides a general spectrophotometric estimate and does not distinguish among individual anthocyanin structures, glycosylated derivatives, acylated forms, or degradation products, all of which can substantially influence antioxidant behavior. In particular, glycosylation may reduce the accessibility of reactive hydroxyl groups and modify the solubility, steric properties, and interactions with radical species. In addition, anthocyanins are strongly affected by environmental conditions and the composition of the extract matrix. We therefore describe this result as an observed association between the TAC and the DPPH activity rather than a direct negative effect of anthocyanins on DPPH scavenging [21].

Notably, cardamom’s antioxidant properties have long been recognized outside the laboratory: it was used in Ayurvedic medicine before the Common Era, mainly for upper respiratory tract conditions (asthma, bronchitis, coughs, tuberculosis), typically administered in the form of an encapsulated powder or pure seeds to chew on. Cardamom was also prescribed for digestive issues: food poisonings, nausea, vomiting, or diarrhea [22]. Its case is a useful reminder that a plant’s traditional therapeutic reputation need not track any single modern assay result.

The CUPRAC parameter shows a very high uniqueness value in the PCA, indicating that its variability is largely independent on the other six tests. *Melissa officinalis*, distinguished by an extremely high content of rosmarinic acid derivatives, achieved an exceptionally high and distinctive CUPRAC result. This suggests that rosmarinic acid, or the synergy among its derivatives, confers a specific copper-reducing potential not explained by general polyphenolic trends or by the quercetin/caffeic acid content alone.

This apparently independent behavior of the CUPRAC can also be explained more generally by its distinct chemical principle. The CUPRAC measures the reduction of Cu(II) ions under near-neutral conditions, whereas the FRAP is based on the reduction of Fe(III) ions under acidic conditions. In contrast, DPPH and ABTS assess the radical-scavenging capacity using different radical probes altogether. Because these methods differ in their oxidant type, pH, redox environment, solvent compatibility, and reaction kinetics, the same extract reasonably produces different responses across assays. Therefore, the behavior of the CUPRAC in our dataset should be interpreted as complementary information about the extracts’ antioxidant profile, rather than as an inconsistency between methods [23].

A high flavonoid content (TFC) constitutes a distinct profile, dominating PCA Component 3 and separating flavonoid-rich samples such as *Crocus sativus* and *Aquilaria crassna* from the rest. Flavonoid activity depends on the hydroxylation pattern and the presence of double bonds that facilitate electron delocalization. However, a high TFC predicts a high overall ranking, without translating into dominance in reduction assays as reliably as the overall TPC does.

More specifically, the key structural factors governing flavonoid antioxidant activity include the number and position of hydroxyl groups, particularly the presence of an ortho-dihydroxy arrangement in the B ring; the presence of a C2=C3 double bond conjugated with a 4-oxo group; and the degree of glycosylation. In general, conjugation and the 4-oxo group enhance the electron delocalization and stabilization of flavonoid radicals, whereas glycosylation may reduce activity by limiting access to reactive sites. However, these effects remain compound-specific and may differ depending on the assay conditions and composition of the extract matrix [24].

The most notable result of this study is the total antioxidant capacity of *Pyrola rotundifolia*, which achieved the highest score in three of the seven conducted tests (TPC, FRAP, ABTS) with significantly higher values than the rest of the studied plants. Different *Pyrola* species have long been used in Traditional Chinese Medicine: Yang et al. described 33 traditional herbal mixtures containing *Pyrola*, mostly administered as decoctions, used principally for anti-arthritic effects (alleviating joint and bone pain), as well as stopping bleeding or lowering blood pressure [25]. Kosuge et al. similarly described *Pyrola rotundifolia* as a TCM drug from the Qu-feng-shi-yao group, used mostly in arthritic diseases, as well as gastric and pulmonary hemorrhages [26]. The literature on *P. rotundifolia* is sparse, given its strong antioxidant properties. The existing studies confirm the presence of antioxidant flavonoids and phenols in *P. rotundifolia*-derived oil [27], and a UHPLC-DAD-MS analysis distinguished 23 compounds (eight unidentified), mostly galloylglucose isomers/derivatives, as well as quercetin derivatives, with a somewhat lower TPC, TFC, and gallotanin content than in our sample [28]. In their review, Yang et al. described the available data on different *Pyrola* species, noting the presence of numerous antioxidant compounds, such as flavonoids, phenol glycosides, quinones, terpenoids, and more [25]. Chang and Inui in 2005 additionally isolated two new glycosides, homoarbutin and isohomoarbutin, with confirmed antimicrobial activity against Gram-positive bacteria [29]. Yang et al. described the high antioxidant activity of a *Pyrola* extract against H_2_O_2_-induced cytotoxicity in neural PC12 cells [30]. These findings are consistent with ours and support *P. rotundifolia*’s potential as an antioxidant therapeutic.

*Zataria multiflora* had the most diverse profile among those studied, containing rosmarinic acid derivatives, caffeic acid derivatives, tannins, and luteolin. This diversity translated into a consistently high activity in most tests.

*Salvia officinalis* combined high levels of luteolin derivatives, rosmarinic acid, and carnosol—a combination that made *S. officinalis* the most effective of all 10 plants in direct free radical scavenging in the DPPH assay.

*Crocus sativus* and *Aquilaria crassna* were the leaders in flavonoid content, but via different compounds. *C. sativus* relied on kaempferol derivatives, while *A. crassna* relied on magniferin derivatives. These distinct profiles clearly separate the two samples from the rest of the extracts in the PCA.

The *Ginkgo biloba* extract demonstrated robust DPPH activity, consistent with a balanced blend of catechins, quercetin, and kaempferol, confirming the role of quercetin and catechins in direct free radical neutralization.

*Paeonia radix* was uniquely rich in iridoids and tannins. Although iridoids are not classic phenolic antioxidants, *P. radix* showed decent activity in the ABTS assay, likely attributable to its tannin and gallic acid content.

*Fragaria x ananasa* contained significant amounts of ellagic acid derivatives and anthocyanins. It demonstrated good activity in both DPPH and ABTS, confirming the berry’s capacity to neutralize oxidative stress.

Cardamom’s case is discussed in detail in previous paragraphs, and its traditional use is noted there as well. Although it contains quercetin and caffeic acid, its very low DPPH activity suggests that other forms—glycosidic or anthocyanin-bund—predominate in this extract, creating a steric barrier that hinders reaction with the neutral radical.

## 4. Materials and Methods

### 4.1. Laboratory Assessments

All dried plant material (DPM) was obtained through commercial sources. *Ginkgo biloba* leaves (batch nr ZMIL1125Z), *Melissa officinalis* leaves (batch nr ZMIL1225Z), and *Salvia officinalis* leaves (batch nr ZSZL1125Z) were purchased from PERSEA Ltd. (Warsaw, Poland); *Crocus sativus* stigmas L. was from HerbaNordPol-Gdańsk Ltd. (Nowy Staw, Poland, batch nr 624012026, grade I (ISO 3632)); *Paeonia Radix* root was from C.M.C (Brzeg, Polska, batch nr 01.2028); *Fragaria* fruit was from PPHU “AWB” Alina Becla (Handzlówka, Poland, batch nr 12.02.2027); *Zataria multiflora* leaves were from Salam Atar (Tehran, Iran, batch nr 21017747); *Pyrola rotundifolia* leaves were from AGREST Ltd. (Lublin, Poland, batch nr 20240930); *Aquilaria crassna* leaves were from Grandawood Agarwood (Brisbane, Australia, expiration date 11/2026*); and *Elettaria Cardamomum* seeds were from YANGO Ltd. (Warsaw, Poland, batch nr P4222.01). *batch number unavailable.

#### 4.1.1. Extract Preparation

The extractions were conducted according to the method described by Kosewski et al. [31]. Ten milliliters of 80% (*v*/*v*) acidified methanol (80% methanol (POCH, Gliwice, Poland) + 20% distilled water + 0.1% HCl (POCH, Poland)) was added to 1 g of DPM crushed in a mortar in the test tube. The tubes were extracted in the ultrasonic bath (InterSonic, Olsztyn, Poland, IS-4) at 35kHz for 3 min at 30 °C ± 5 °C. They were then centrifuged at 3000 rpm for 10 min at 25 °C (Jouan B4i, Thermo Fisher, Waltham, MA, USA. The supernatant was removed, and the solid phase underwent a similar second extraction. Finally, the supernatants were pooled and filtered for further analysis. From each plant sample, one extract was prepared.

#### 4.1.2. Total Phenolic Content Assessment (TPC)

The concentrated Folin–Ciocalteu reagent (Sigma Aldrich, Buchs, Switzerland) was dissolved in distilled water in a 1:10 proportion. The 7.5% Na_2_CO_3_ (Chempur, Piekary Śląskie, Poland) solution in acidified methanol (0.1% HCl) was prepared. Into a 96-well plate, 20 μL of the extract was added, followed by 100 μL of the Folin–Ciocalteu solution. After 3 min, 80 μL of the Na_2_CO_3_ solution was added. The plate was then shaken and incubated for 60 min at room temperature in the dark. The absorbance was measured at λ = 765 nm. The total phenolic content was expressed as mg of gallic acid equivalent per g of dried plant material (mg GAE/g DPM) using a calibration curve for gallic acid (Chempur, Poland) (50–500 μg/mL). The assessment was conducted in 3 technical replicates.

#### 4.1.3. Total Flavonoid Content Assessment (TFC)

A solution of 2% AlCl_3_ (Chempur, Poland) in acidified methanol (0.1%HCl) was prepared. Into a 96-well plate, 20 μL of the extract and 100 μL of the AlCl_3_ solution were added. The plate was then shaken and incubated for 10 min at room temperature in the dark. The absorbance was measured at λ = 415 nm. The total flavonoid content was expressed as the quercetin equivalent per g of dried plant material (QE/g DPM) using a calibration curve for quercetin (Sigma Aldrich, Bengaluru, India) (20–240 μg/mL). The assessment was conducted in 3 technical replicates.

#### 4.1.4. Total Anthocyanin Content Assessment (TAC)

Nineteen milliliters of methanol was added to 1 g of dried and crushed plant material. The samples were shaken for 30 min in darkness and then filtered through a paper filter into a 20 mL measuring flask. The filtrates were made up to 20 mL with methanol to achieve the basic extract, which was later diluted in a 1:50 proportion with acidified methanol (0.1% HCl). The absorbance was measured on a 96-well plate, at a wavelength of 528 nm with an additional measurement at 700 nm as a correction for opacity. The total anthocyanin content was expressed as mg of cyanidin-3-O-glucoside equivalent per g of dried plant material (mg cyanidin-3-O-glucoside/g DPM). The assessment was conducted in 3 technical replicates.

#### 4.1.5. DPPH Radical Reduction Method

A 0.1 mM DPPH (Sigma Aldrich, Steinheim, Germany) stock was prepared by dissolving 3.94 mg of DPPH in 100 mL of acidified methanol (0.1% HCl). The absorbance of the stock was measured in λ = 517 nm. To the 96-well plate, 20 μL of the extract and 180 μL of the DPPH solution were added. The plate was then shaken and incubated for 30 min at room temperature in the dark. The absorbance was measured in λ = 517 nm. The ability to reduce the DPPH radical was expressed as mg of Trolox (Sigma Aldrich, Moscow, Russia) equivalent per g of dried plant material (mg TE/g DPM) using a calibration curve for Trolox (25–200 µM). The assessment was conducted in 3 technical replicates.

#### 4.1.6. Quenching of the Characteristic Absorption Band of the ABTS•+ Cation Radical

A 7 mM ABTS•+ stock was prepared by dissolving 38.4 mg of ABTS (Sigma Aldrich, Saint Louis, MO, USA) diammonium salt (2,2′-azino-bis(3-ethylbenzothiazoline-6-sulfonic acid)) in 10 mL of deionized water. A 2.45 mM potassium persulfate was made by dissolving 5.59 mg of K_2_S_2_O_8_ (Chempur, Poland) in 10 mL of deionized water. A total of 5 mL of each solution was mixed and incubated for 16 h in darkness at room temperature. The prepared mixture was then diluted in acidified methanol (0.1% HCl) so that the absorbance at 734 nm was ~0.700 ± 0.05. Into a 96-well plate, 10 µL of the plant extract and 200 µL of the ABTS•+ solutions were added, and the plate was then shaken and incubated for 6 min at room temperature. The absorbance was measured at 734 nm. Quenching of the characteristic absorption band of the ABTS•+ cation radical was expressed as mg of Trolox equivalent per g of dried plant material (mg TE/g DPM) using a calibration curve for Trolox (50–350 µM). The assessment was conducted in 3 technical replicates.

#### 4.1.7. Ferric Reducing Antioxidant Power (FRAP)

A 300 mM acetate buffer (pH = 3.6) was prepared by mixing 3.09 g of sodium acetate in 80 mL of deionized water, followed by 17 mL of concentrated acetic acid, and the solution was then mixed. The pH was brought to 3.6, and the solution was made up to 100 mL with deionized water. A 10 mM 2,4,6-tripyridyl-s-triazine (TPTZ) (Sigma Aldrich, Switzerland) in 40 mM HCl was made by dissolving 31.2 mg of TPTZ in 8 mL of 40 mM HCl. An amount of 43,2 mg of FeCl_3_·6H_2_O (Chempur, Poland) was dissolved in 8 mL of deionized water to prepare 20 mM FeCl_3_. Then, the acetic buffer, TPTZ, and FeCl_3_ were mixed in a 10:1:1 proportion to make the FRAP stock. Into a 96-well plate, 20 μL of the extract and 180 μL of the stock were added, which was then incubated for 30 min in 37 °C in the dark. The absorbance was measured at λ = 593 nm. The ferric reducing antioxidant power was expressed as mg of Trolox equivalent per g of dried plant material (mg TE/g DPM) using a calibration curve for Trolox (12.51–93.86 µg/mL). The assessment was conducted in 3 technical replicates.

#### 4.1.8. Cupric Ion Reducing Antioxidant Capacity (CUPRAC)

The following solutions were prepared: 10 mM CuCl_2_•2H_2_O (Chempur, Poland), ammonium acetate buffer (pH = 7), and 7.5 mM neocuproine (2,9-dimethyl-1,10-phenanthroline) (Sigma Aldrich, Switzerland) in ethanol. The CUPRAC reagent was prepared by mixing 50 µL of CuCl_2_·2H_2_O, 50 µL of neocuproine, and 60 µL of NH_4_Ac. For testing, 40 µL of the plant extract and 160 µL of the CUPRAC solution were mixed on a 96-well plate. The plate was then shaken and incubated for 30 min at room temperature in the dark. The absorbance was measured at 450 nm. The cupric ion reducing antioxidant capacity was expressed as mg of Trolox equivalent per g of dried plant material (mg TE/g DPM) using a calibration curve for Trolox (12.51–125.15 µg/mL). The assessment was conducted in 3 technical replicates.

All spectrophotometric measurements were made using a Thermo Scientific (Waltham, MA, USA) Multiscan GO spectrophotometer. All measurements were conducted according to the authors’ original methodology.

#### 4.1.9. Rationale for the Combined Use of Assays

The methods applied in this study can be divided into two groups: compositional measurements and antioxidant activity assays. The total phenolic content, measured using the Folin–Ciocalteu method, provides an operational estimate of phenolic-reactive and other reducing compounds and is therefore not fully specific to phenolic compounds. The total flavonoid content, determined using the aluminum chloride method, depends on the ability of selected flavonoid structures to form complexes with aluminum ions. The total anthocyanin content provides an estimate of anthocyanin pigments, commonly expressed as cyanidin-3-glucoside equivalents. Therefore, these methods describe different aspects of extract composition rather than direct antioxidant activity alone [21,32,33].

The DPPH and ABTS assays measure the radical-scavenging capacity; however, they differ in the radical species used and in the reaction environment. The DPPH assay employs a stable nitrogen-centered radical and is typically performed in organic solvent systems. In contrast, the ABTS assay uses the ABTS radical cation and may be applied to both hydrophilic and lipophilic antioxidant compounds. As a result, the two methods may respond differently to the same extract and provide complementary information on the radical-scavenging capacity [34,35].

The FRAP and CUPRAC measure the reducing capacity, but they differ in the metal-ion probes and reaction conditions used. The FRAP operates under acidic conditions and is based on the reduction of the Fe(III)-TPTZ complex, whereas the CUPRAC uses the reduction of a Cu(II)-neocuproine complex under near-neutral conditions. These methodological differences can result in different sensitivities toward individual phenolic compounds and other reducing substances present in the extracts [18,23].

For these reasons, the combined use of the TPC, TFC, total anthocyanin content, DPPH, ABTS, FRAP, and CUPRAC enables a broader assessment of both the chemical composition and the antioxidant properties of the extracts. Each method reflects a different chemical aspect of antioxidant potential, including radical scavenging, the metal-ion reducing capacity, and the abundance of selected groups of phenolic compounds. We also clarify that these assays represent in vitro chemical measurements and should not be interpreted as direct evidence of antioxidant effects under physiological or in vivo conditions [36].

### 4.2. HPLC Analysis

Flavonoids and their glycosides were determined after alkaline and/or acidic hydrolysis to release aglycones. Quantitative analyses were performed using an Acquity H-Class UPLC^®^ system (Waters, Milford, MA, USA) equipped with a PDA detector, and LC-MS detection was also used for structural confirmation. Chromatographic separation was performed on an Acquity UPLC^®^ BEH C18 column (100 × 2.1 mm, 1.7 μm; Waters, Wexford, Ireland).

Proanthocyanidins were determined after sonication-assisted methanol–water extraction (70:30, *v*/*v*). Analyses were performed using the UPLC-PDA method on an Acquity H-Class system (Waters, USA) with an Acquity UPLC^®^ BEH C18 column (100 × 2.1 mm, 1.7 μm). Gradient elution was performed using mobile phases A (acetonitrile + 0.1% formic acid) and B (water + 0.1% formic acid). Detection was performed at λ = 280 nm. Identification was based on a comparison of the spectra and retention times with catechin and procyanidin standards, and quantification was performed using a calibration curve. The limit of quantification was 1 μg/g.

Phenolic acids were determined after extraction combined with acid–base hydrolysis. Analyses were performed using an Acquity H-Class UPLC system (Waters, USA) with a PDA detector and an Acquity UPLC^®^ BEH C18 column (100 × 2.1 mm, 1.7 μm). LC-MS detection was also used for structural confirmation. Elution was carried out using a gradient system: phase A—acetonitrile with 0.1% formic acid, phase B—water with 1% formic acid (pH = 2). Detection was performed at λ = 320 nm. Identification was based on a comparison of the retention times with standards, and quantification was performed using the external standard method. The limit of quantification was 1 μg/g.

Bilobalide and ginkgolides A, B, and C were determined by UPLC-PDA, and LC-MS detection was also used for structural confirmation. The analyses were performed on an Acquity H-Class system (Waters, USA) with an Acquity UPLC^®^ BEH C18 column (100 × 2.1 mm, 1.7 μm). Gradient elution was performed using mobile phases A (acetonitrile + 0.1% formic acid) and B (water + 0.1% formic acid). PDA detection was performed at λ = 220 nm. Identification was based on retention times and comparison with reference standards. Quantification was performed using a calibration curve. The limit of quantification was 1 μg/g [37,38].

Each analysis was conducted in 3 technical replicates.

### 4.3. Statistical Analysis

We assessed the correlation between the content of polyphenols, flavonoids and anthocyanins, and the antioxidant capacity test results (DPPH, ABTS, FRAP, CUPRAC). To achieve this, we created a correlation matrix and calculated Spearman’s rho and *p*-value (*p* < 0.05 was considered statistically significant). We also conducted a principal component analysis (PCA) based on the eigenvalues, and applied varimax rotation.

To compare the differences between the plants’ scores in each of the seven tests, we performed ANOVA tests. Firstly, we checked for normality using the Shapiro–Wilk test. In case of a normal distribution, we followed with a parametric ANOVA, assessing the appropriateness of either Welch’s or Fisher’s adjustment via Leven’s test. We used Games–Howell’s or Tukey’s post hoc test, respectively. In the case of a non-normal distribution, we followed with the Kruskal–Wallis test and Dwass–Steel–Critchlow–Flinger test as a post hoc.

To assess the potential correlation between the composition of extracts determined by HPLC and their antioxidant properties, we created a heatmap and relationship graphs.

All statistical analyses were performed using jamovi (version 2.7, 2025) and R (version 4.5.3).

## 5. Conclusions

Our analysis shows that a plant’s antioxidative potential is not a one-dimensional trait, but rather a result of specific relationships between their chemical profile and mechanisms of action in a given environment. This study shows a strong correlation between the ability to neutralize free radicals and molecular structure. The key factor is the density of hydroxyl groups and the presence of specific structures, such as catechol groups in the main bioactive compounds. The more robust the above-mentioned structures, the easier it is to donate electrons, which directly translates to the reductive potential.

One of the most important findings is the steric hindrance hypothesis. We conjecture that a high concentration of a given compound (e.g., anthocyanins) does not guarantee high values in all antioxidant capacity tests. It is probable that large glycoside compounds may come across physical barriers, which reduce the contact with specific radicals. That would mean that some plants can possess a very high effectiveness in one antioxidant capacity test while being almost passive in others.

The PCA enables the classification of plants based on their unique antioxidant niche, rather than a single high result. While some species dominate direct radical scavenging, others show the ability to chelate metals or reduce ions. This suggests that, in preventative health care, the diversity of phytocompound sources may be as important as any single plant’s peak performance.

Our results constitute biochemical justification for nutritional interventions such as the MIND diet, showing that specific polyphenols not only reduce oxidative stress, but may also stimulate cells’ endogenous defensive mechanisms. However, these assays represent in vitro chemical measurements and should not be interpreted as direct evidence of antioxidant effects under physiological or in vivo conditions.

It is worth emphasizing that all the described plants have been used as food additives and traditional medicinal plants for years, and represent a potential source of antioxidants relevant to chronic diseases. Neuroprotective and neuro-regenerative properties of plant extracts are being increasingly considered in a field of modern therapeutic research. Despite promising results, the literature remains sparse, and translating these findings into therapy will require further in vitro and in vivo research—particularly to establish precise mechanisms of action and to characterize the bioavailability and safety of these phytocompounds in vivo. Fully realizing plants’ therapeutic potential could bring forward novel therapies for currently incurable diseases. Our results serve both as an in-depth explanation of known mechanisms and as a signpost for future research.

## Figures and Tables

**Table 1:** Total antioxidant capacity of plant extracts.

	TPC [mg GAE/g DPM]	TFC [mg QE/g DPM]	TAC [mg Cyanidin-3-O-glucoside/g DPM]	FRAP [mg TE/g DPM]	ABTS [mg TE/g DPM]	DPPH [mg TE/g DPM]	CUPRAC [mg TE/g DPM]
*M. officinalis*	5.77	5.83	0.4327	5.25	60.43	31.96	136.81
*S. officinalis*	10.54	44.36	0.0843	7.63	86.71	101.76	94.00
*P. rotundifolia*	55.55 *	87.72	0.1392	41.62 ***	108.04 ***	74.27	79.95
*C. sativus*	7.86	206.20	0.0928	8.11	40.38	52.58	92.57
*A. crassna*	20.45 *	167.79	0.1216	24.04	22.98	80.40	93.53
*E. cardamomum*	0.72	0.61	0.4639 *	0.45	5.41	0.24	5.36
*F. x ananassa*	6.45	3.59	0.0398	5.44	61.40	75.69	51.90
*G. biloba*	7.23	26.73	0.0795	2.65	50.14	81.50	43.00
*P. radix*	6.09	2.87	0.0620	9.40	73.48	55.44	70.32
*Z. multiflora*	14.20 *	48.63	0.1704	10.02	82.29	57.25	83.95

* *p* < 0.05. *** *p* < 0.001.

**Table 2:** Correlation between TPC, TFC, and TAC content and antioxidant capacity.

		TPC [mg GAE/g DPM]	TFC [mg QE/g DPM]	TAC [mg/g DPM]
FRAP [mg TE/g DPM]	Spearman’s rho	0.818 **	0.648 *	−0.042
	*p*-value	0.007	0.049	0.919
ABTS [mg TE/g DPM]	Spearman’s rho	0.442	0.103	−0.224
	*p*-value	0.204	0.785	0.537
DPPH [mg TE/g DPM]	Spearman’s rho	0.576	0.309	−0.576
	*p*-value	0.088	0.387	0.088
CUPRAC [mg TE/g DPM]	Spearman’s rho	0.333	0.527	0.200
	*p*-value	0.349	0.123	0.584

* *p* < 0.05. ** *p* < 0.01.

**Table 3:** Principal component analysis (PCA).

	Component			
	1	2	3	Uniqueness
TPC [mg GAE/g DPM]	0.947			0.0914
TFC [mg QE/g DPM]	0.325		0.906	0.0363
TAC [mg/g DPM]		−0.950		0.0780
FRAP [mg TE/g DPM]	0.936			0.0865
ABTS [mg TE/g DPM]	0.608	0.465	−0.566	0.0933
DPPH [mg TE/g DPM]		0.917		0.0935
CUPRAC [mg TE/g DPM]	0.457			0.7488

**Table 4:** Eigenvalue and (cumulative) percentage of variance.

	Eigenvalue	% of the Variance	Cumulative %
Dim. 1	3.24904	46.4148	46.4
Dim. 2	1.35553	19.3647	65.8
Dim. 3	1.16766	16.6808	82.5

**Table 5:** Percentage of groups of chemical compounds detected in HPLC.

Plant	Group of Compounds	Percentage Share (%)
*Melissa officinalis*	Rosmarinic acid derivatives	77.82%
	Caffeic acid derivatives	19.93%
	Gallic acid derivatives	0.01%
	Luteolin derivatives	2.23%
*Salvia officinalis*	Rosmarinic acid derivatives	28.93%
	Caffeic acid derivatives	0.06%
	Carnosol derivatives	24.78%
	Luteolin derivatives	40.74%
	Apigenin derivatives	2.21%
	Hispidulin derivatives	3.27%
	Quercetin derivatives	0.01%
*Pyrola rotundifolia*	Quercetin derivatives	57.52%
	Gallic acid derivatives	25.17%
	Catechin derivatives	0.28%
	Iridoids	17.03%
*Crocus sativus*	Gallic acid derivatives	27.80%
	Kaempferol derivatives	72.20%
*Aquilaria crassna*	Mangiferin derivatives	94.90%
	Genkwanin derivatives	5.10%
*Elettaria cardamomum*	Caffeic acid derivatives	39.13%
	Gallic acid derivatives	12.61%
	Catechin derivatives	32.28%
	Quercetin derivatives	11.30%
	Kaempferol derivatives	2.39%
	Flavanone derivatives	2.28%
*Fragaria x ananasa*	Ellagic acid derivatives	49.71%
	Anthocyanins	27.47%
	Gallic acid derivatives	2.87%
	Caffeic acid derivatives	10.95%
	Catechin derivatives	6.48%
	Quercetin derivatives	1.43%
	Kaempferol derivatives	1.09%
*Ginkgo biloba*	Quercetin derivatives	33.31%
	Kaempferol derivatives	21.35%
	Catechin derivatives	37.81%
	Caffeic acid derivatives	0.73%
	Gentisic acid derivatives	0.84%
	Other diterpenes	5.95%
*Paeonia radix*	Iridoids	81.30%
	Tannins	14.32%
	Gallic acid derivatives	2.81%
	Caffeic acid derivatives	0.85%
	Catechin derivatives	0.73%
*Zataria multiflora*	Rosmarinic acid derivatives	31.96%
	Caffeic acid derivatives	24.40%
	Luteolin derivatives	5.87%
	Flavanones	1.70%
	Tannins	25.06%
	Apigenin derivatives	4.53%
	Quercetin derivatives	4.24%
	Kaempferol derivatives	2.24%

## Data Availability

The dataset is available upon request.

## Supplementary Materials

The following supporting information can be downloaded at: https://www.mdpi.com/article/10.3390/molecules31142523/s1, Table S1: Chemical composition of studied plants detected in HPLC.

## Abbreviations

The following abbreviations are used in this manuscript:

PCA	Principal component analysis
NOS	Nitric oxide synthase
HAT	Hydrogen atom transfer
SET	Single electron transfer
ROS	Reactive oxygen species
DPM	Dried plant material
DE	Dried extract
TPC	Total phenolic content
TFC	Total flavonoid content
ABTS	Quenching of the characteristic absorption band of the ABTS•+ cation radical
DPPH	DPPH radical reduction method
FRAP	Ferric reducing antioxidant power
CUPRAC	Cupric ion reducing antioxidant capacity
HPLC	High-performance liquid chromatography
UPLC	Ultra-high-performance liquid chromatography
PDA	Photodiode array
MS	Mass spectrometry
TE	Trolox equivalent
GAE	Gallic acid equivalent
QE	Quercetin equivalent
MIND	Mediterranean–DASH Intervention for Neurodegenerative Delay
NF-κB	Nuclear factor kappa-light-chain enhancer of activated B cells
Nrf2/ARE	Nuclear factor erythroid 2-related factor 2/antioxidant response element
PC12	Rat pheochromocytoma cells
TCM	Traditional Chinese Medicine
