# Supplementary material for: Total Polyphenol Content and Other Antioxidant Capacity Indicators in Selected Edible Plants and Herbs
Source: Molecules. 2026 Jul 20;31(14):2523. doi: 10.3390/molecules31142523 (PMC13415093; doi:10.3390/molecules31142523)
Supplement: Supplementary file 1 [file molecules-31-02523-s001.zip › molecules-4348217-supplementary.pdf]

Table S1. Chemical composition of studied plants, detected in HPLC.

Plant	Phenolic compound	Concentration (mg/g DE)	Group of compounds	Total concentration (mg/g DE)
Melissa officinalis	Rosmarinic acid	18,4	Rosmarinic acid derivatives	327,5
	Caffeic acid	1,79	Caffeic acid derivatives	83,89
	Protocatechuic acid	0,05	Gallic acid derivatives	0,05
	Rosmarinic acid	200,1	Luteolin derivatives	9,4
	Rosmarinic acid	34,5		
	Rosmarinic acid	74,5		
	Neochlorogenic acid	44,5		
	Sum of caffeic acid derivatives	32,5		
	Luteolin-7-O-glucuronide	9,4		
	Chlorogenic acid	5,1		
Salvia officinalis	Rosmarinic acid (trans)	77,4	Rosmarinic acid derivatives	106,12
	Rosmarinic acid (cis)	0,92	Caffeic acid derivatives	0,21
	Rosmarinic acid	18,5	Carnosol derivatives	90,9
	Chlorogenic acid	0,01	Luteolin derivatives	149,4
	Caffeic acid	0,02	Apigenin derivatives	8,09
	Ferulic acid	0,15	Hispidulin derivatives	12
	p-Coumaric acid	0,02	Quercetin derivatives	0,04
	Caffeoylquinic acid derivative	0,01		
	Sageric acid	2,8		
	Salvianolic acid K	6,5		
	Carnosic acid	44,3		
	Carnosol	32,3		
	Rosmadial	6,9		
	Methylcarnosate	7,4		
	Luteolin-7-O-glucuronide	80,4		
	Luteolin-7-O-glucoside	35,1		
	Luteolin acetylglucoside	14,5		
	Luteolin diglucuronide	10,2		
	Luteolin-7-O-rutinoside	6,8		
	6-Hydroxyluteolin-7-O-glucuronide	2,4		
	Apigenin-7-O-glucoside	5,8		
	Apigenin	2,1		
	Apigenin	0,19		

	Hispidulin	1,8		
	Glucuronide hispidulin	10,2		
	Quercetin-7-O-glucoside	0,04		
Pyrola rotundifolia	Quercetin O-galloylhexoside	25,4	Quercetin derivatives	38,84
	Galloylglucose isomer I	13,1	Gallic acid derivatives	17
	Monotropein	11,5	Catechin derivatives	0,19
	Hyperoside (quercetin-3-O-galactoside)	10,5	Iridoids	11,5
	Galloshikic acid	1,16		
	Guajaverine (quercetin-3-O-arabinopyranoside)	0,79		
	6-O-galloylhomoarbutin	1,1		
	Quercetin O-galloylpentoside	0,82		
	Digalloylglucose	0,91		
	Isoquercitrin (quercetin-3-O-glucoside)	0,68		
	Galloylglucose isomer II	0,73		
	Quercetin-2''-O-galloylgalactoside	0,65		
	Epicatechin gallate	0,19		
Crocus sativus	Gallic acid	2,1	Gallic acid derivatives	3,58
	Pyrogallol	1,48	Kempferol derivatives	9,3
	Kaempferol-3-O-sophoroside	9,3		
Aquilaria crassna	Mangiferin	155,1	Mangiferin derivatives	376,2
	Iriflophenone 3,5-C-β-D-diglucoside	116,3	Genkwanin derivatives	20,2
	Iriflophenone 3-C-β-D-glucoside	71,3		
	Iriflophenone 2-O-α-rhamnoside	33,5		
	Genkwanin 5-O-β-primeveroside	10,2		

	Genkwanin 4′-methyl ether 5-O-β-primeveroside	7,6		
	Genkwanin	2,4		
Elletaria cardamomum	Gallic acid	1,16	Caffeic acid derivatives	3,6
	Caffeic acid	0,76	Gallic acid derivatives	1,16
	Chlorogenic acid	1,44	Catechin derivatives	2,97
	p-Coumaric acid	0,58	Quercetin derivatives	1,04
	Ferulic acid	0,82	Kempferol derivatives	0,22
	Catechin	1,66	Flavanone derivatives	0,21
	Epicatechin	1,31		
	Rutin	0,58		
	Quercetin	0,46		
	Kempferol	0,22		
	Naringenin	0,21		
Fragaria x ananasa	Ellagic acid	57,2	Ellagic acid derivatives	145,7
	Ellagotanins (total)	88,5	anthocyanins	80,5
	Gallic acid	8,4	gallic acid derivatives	8,4
	Chlorogenic acid	21,6	caffeic acid derivatives	32,1
	Caffeic acid	6,3	catechin derivatives	19
	p-coumaric acid	4,2	quercetin derivatives	4,2
	Catechin	8,4	kaempferol derivatives	3,2
	Epicatechin	10,6		
	Quercetin	4,2		
	Kaempferol	3,2		
	pelargonidin-3-glucoside	33		
	cyanidin-3-glucoside	47,5		
Ginkgo biloba	Quercetin	2,5	Quercetin derivatives	18,15
	Rutin (quercetin-3-O-rutinoside)	12,5	Kempferol derivatives	11,63
	Isoquercitrin (quercetin-3-O-glucoside)	1,5	Catechin derivatives	20,6
	Quercetin (quercetin-3-O-rhamnoside)	0,7	Caffeic acid derivatives	0,4
	Hyperoside (quercetin-3-O-galactoside)	0,04	Gentisic acid derivatives	0,46
	Quercetin-3-O-arabinoside	0,88	Other diterpenes	3,24

	Quercetin-3-O-xyloside	0,03		
	Afzelin (kaempferol-3-O-rhamnoside)	1,5		
	Astragaline (kaempferol-3-O-glucoside)	0,4		
	Kaempferol-3-O-rutinoside	1,7		
	Kaempferol-3-O-galactoside	6,1		
	Kaempferol-3-O-arabinoside	1,33		
	Kaempferol-3-O-xyloside	0,6		
	Proanthocyanidins	20,6		
	Gentisic acid	0,46		
	Acid p-Coumaric Acid	0,23		
	Caffeic Acid	0,17		
	Bilobalide	0,44		
	Ginkgolic Acid	0,01		
	Ginkgolide A	0,8		
	Ginkgolide B	1,1		
	Ginkgolide C	0,89		
Paeonia radix	Paeoniflorin	110,4	Iridoids	157,6
	Albiflorin	37,8	Tannins	27,76
	Benzoylpaeoniflorin	9,4	Gallic acid derivatives	5,44
	Galloylpaeoniflorin	7,66	Caffeic acid derivatives	1,64
	Gallic acid	4,32	Catechin derivatives	1,42
	Protocatechuic acid	1,12		
	Caffeic acid	1,05		
	p-Coumaric acid	0,59		
	Catechin	1,04		
	Epicatechin	0,38		
	Hydrolyzable tannins	20,1		
Zataria multiflora	Rosmarinic acid	22,7	Rosmarinic acid derivatives	22,7
	Chlorogenic acid	9,4	Caffeic acid derivatives	17,33
	Caffeic acid	5,52	Luteolin derivatives	4,17
	Ferulic acid	1,42	Flavanones	1,21
	p-coumaric acid	0,99	Tannins	17,8
	Luteolin	4,17	Apigenin derivatives	3,22
	Apigenin	3,22	Quercetin derivatives	3,01
	Quercetin (glycosides)	3,01	Kempferol derivatives	1,59

	Kaempferol (glycosides)	1,59
	Naringenin	1,21
	Total tannins	17,8
